# ^125^I-labelled human chorionic gonadotrophin (hCG) as an elimination marker in the evaluation of hCG decline during chemotherapy in patients with testicular cancer

**DOI:** 10.1038/sj.bjc.6690565

**Published:** 1999-07

**Authors:** T B Christensen, F Engbaek, J Marqversen, S I Nielsen, C Kamby, H von der Maase

**Affiliations:** Departments of Oncology, Clinical Physiology and Nuclear Medicine and Clinical Biochemistry, Aarhus University Hospital, Denmark; Departments of Oncology and Clinical Physiology and Nuclear Medicine, Herlev Hospital, University of Copenhagen, Denmark

**Keywords:** gonadotrophin, human chorionic, testicular neoplasm, chemotherapy

## Abstract

The rate of reduction in the concentration of serum human chorionic gonadotrophin (hCG) following chemotherapy for germ cell tumours may follow a complex pattern, with longer apparent half-life during later stages of chemotherapy, even in patients treated successfully. The commonly used half-life of less than 3 days for hCG to monitor the effect of chemotherapy in patients with germ cell tumours of the testis may represent too simple a model. ^125^I-labelled hCG was injected intravenously in 27 patients with germ cell tumours and elevated hCG during chemotherapy. The plasma radioactivity and hCG concentrations were followed. During chemotherapy, the plasma disappearance of hCG showed a biphasic pattern, with an initial fast and a later slow component in all patients. Using the steep part of the hCG plasma disappearance curve, five patients who achieved long-term remission had half-lives longer than 3 days (3.6–6.8 days), whereas four out of five patients not achieving long-term remission had half-lives shorter than 3 days. After the third treatment cycle, eight patients who achieved long-term remission had hCG half-lives longer than 3 days (7.4–17.0 days). In these patients, the plasma disappearance of [^125^I]hCG was equivalent to that of hCG. Thus, the slow decline of hCG represented a slow plasma disappearance rather than a hCG production from vital tumour cells and could, consequently, not be used to select patients for additional or intensified chemotherapy. The concept of a fixed half-life for plasma hCG during treatment of hCG-producing germ cell tumours is inappropriate and should be revised. Difficulties in interpreting a slow decline of hCG may be overcome by comparing the plasma disappearance of total hCG with the plasma disappearance of [^125^I]hCG. © 1999 Cancer Research Campaign


*British Journal of Cancer* (1999) **80**(10), 1577–1581
© 1999 Cancer Research Campaign
Article no. bjoc.1999.0565

### $^{125}$I-labelled human chorionic gonadotrophin (hCG) as an elimination marker in the evaluation of hCG decline during chemotherapy in patients with testicular cancer

**TB Christensen[1,5], F Engbaek[3], J Marqversen[2], SI Nielsen[5], C Kamby[4] and H von der Maase[1]**

Departments of [1]Oncology, [2]Clinical Physiology and Nuclear Medicine and [3]Clinical Biochemistry, Aarhus University Hospital, Denmark; Departments of [4]Oncology and [5]Clinical Physiology and Nuclear Medicine, Herlev Hospital, University of Copenhagen, Denmark

**Summary** The rate of reduction in the concentration of serum human chorionic gonadotrophin (hCG) following chemotherapy for germ cell tumours may follow a complex pattern, with longer apparent half-life during later stages of chemotherapy, even in patients treated successfully. The commonly used half-life of less than 3 days for hCG to monitor the effect of chemotherapy in patients with germ cell tumours of the testis may represent too simple a model. $^{125}$I-labelled hCG was injected intravenously in 27 patients with germ cell tumours and elevated hCG during chemotherapy. The plasma radioactivity and hCG concentrations were followed. During chemotherapy, the plasma disappearance of hCG showed a biphasic pattern, with an initial fast and a later slow component in all patients. Using the steep part of the hCG plasma disappearance curve, five patients who achieved long-term remission had half-lives longer than 3 days (3.6–6.8 days), whereas four out of five patients not achieving long-term remission had half-lives shorter than 3 days. After the third treatment cycle, eight patients who achieved long-term remission had hCG half-lives longer than 3 days (7.4–17.0 days). In these patients, the plasma disappearance of [$^{125}$I]hCG was equivalent to that of hCG. Thus, the slow decline of hCG represented a slow plasma disappearance rather than a hCG production from vital tumour cells and could, consequently, not be used to select patients for additional or intensified chemotherapy. The concept of a fixed half-life for plasma hCG during treatment of hCG-producing germ cell tumours is inappropriate and should be revised. Difficulties in interpreting a slow decline of hCG may be overcome by comparing the plasma disappearance of total hCG with the plasma disappearance of [$^{125}$I]hCG.

**Keywords:** gonadotrophin; human chorionic; testicular neoplasm; chemotherapy

The half-life of hCG during chemotherapy may be used to monitor the effect of chemotherapy in patients with germ cell tumours of the testis (Klein, 1993; Bosl and Motzer, 1997). Thus, Toner et al (1990) evaluated the half-life of hCG in 134 patients with testicular cancer and elevated hCG. All patients received cisplatin-containing chemotherapy. Marker half-life of hCG was deemed to be satisfactory if the half-life of hCG was less than or equal to 3 days. To calculate the half-life, at least two measurements of hCG between day 7 and day 90 from start of chemotherapy were used. The authors concluded that a prolonged half-life of hCG after chemotherapy identifies patients unlikely to achieve a complete remission (CR). In a recent study, Gerl et al (1996) confirmed these findings and concluded that marker half-life analysis complements pretreatment risk stratification and supports selection of patients for early dose-intensified chemotherapy. In a retrospective study, Motzer et al (1992) reported results from the treatment of patients with refractory germ cell tumour with high-dose chemotherapy and autologous bone marrow rescue. Slow marker decline for hCG (> 3 days) after the first two cycles of cisplatin-based chemotherapy was used to identify patients with unsatisfactory response to initial treatment. These patients were changed to high-dose chemotherapy and autologous bone marrow rescue. Accordingly, Murphy et al (1994) reported that the rate of serum decline of hCG during the first two cycles of chemotherapy for advanced germ cell tumour could identify patients who might benefit from an early change to more intensive therapy. The acceptable half-life of hCG was defined as less than 3 days. Bosl and Chaganti (1994) reported that a half-life of hCG longer than 3 days after two cycles of chemotherapy may be used to predict which patients would obtain less than a CR and accordingly would benefit from intensified treatment. Furthermore, Bosl and Motzer (1997) stated that a plateau or slow clearance suggests residual active disease and that persistently elevated serum concentrations of hCG after initial chemotherapy are associated with viable, often unresectable, residual germ cell tumours and should be treated with additional chemotherapy rather than surgical resection.

On the other hand, patients with a half-life of hCG longer than 3 days during chemotherapy may achieve CR and long-time survival (Horwich and Peckham, 1984; Andreyv et al, 1993; Stevens et al, 1995; de Wit et al, 1997; Zon et al, 1998). Thus, a prolonged half-life and persistence of a high plasma concentration may be due to factors other than release of the hormone from tumour cells. The chemotherapy itself could, in fact, be responsible for a prolonged plasma disappearance of hCG. We decided to compare the plasma disappearance of [$^{125}$I]hCG injected intravenously with the plasma disappearance of total hCG (injected plus tumour-derived hCG) to

*Received 1 April 1998*
*Revised 10 June 1998*
*Accepted 15 June 1998*

*Correspondence to:* TB Christensen, Department of Clinical Physiology and Nuclear Medicine KF4011, Rigshospitalet, Copenhagen DK-2100, Denmark

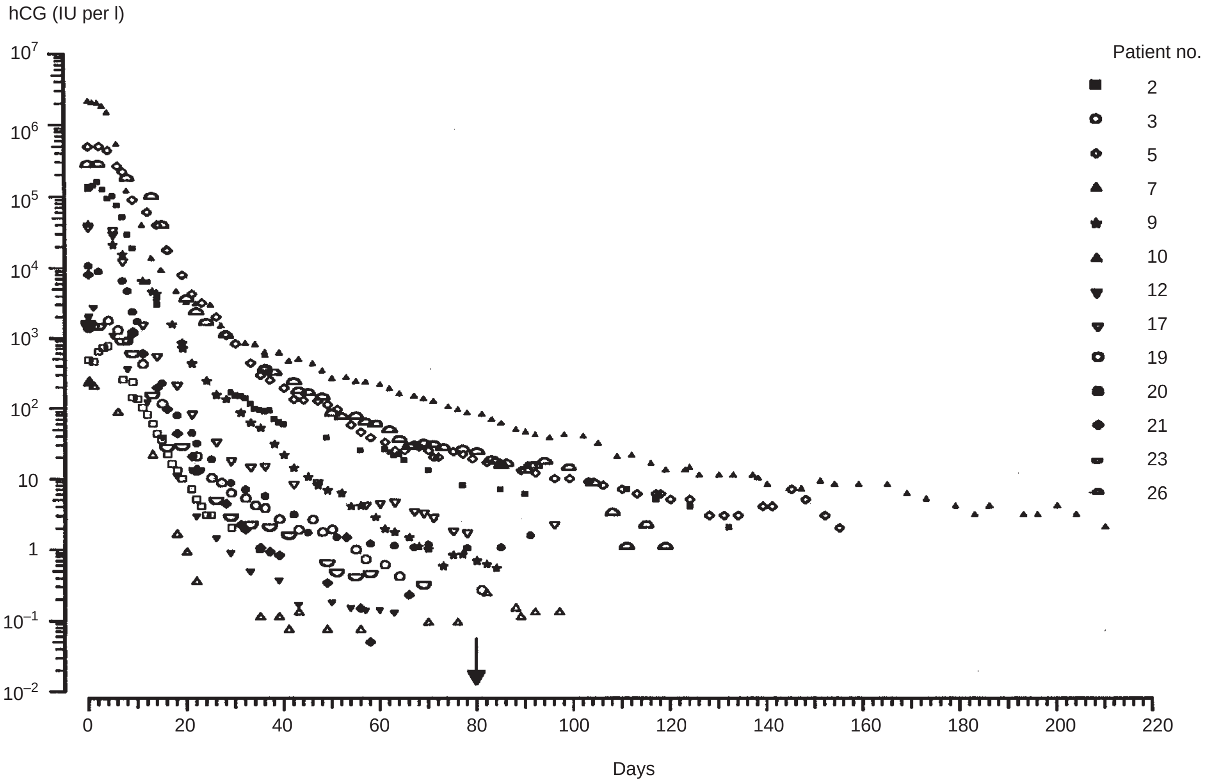

**Figure 1** hCG measured immunologically during and after chemotherapy in 13 patients with initial hCG > 200 IU $l^{-1}$ (median 12 900 IU $l^{-1}$, range 120 000 IU $l^{-1}$) and continuous remission during follow-up. Arrow indicates completion of the last, i.e. the fourth, cycle of chemotherapy. All patients were alive without evidence of disease after a median period of 24+ months (range 8+ to 44+ months) after completion of chemotherapy visualize the disappearance of hCG in patients with germ cell tumours of the testis receiving chemotherapy.

### MATERIALS AND METHODS

In the period from March 1994 to June 1997, patients with advanced germ cell tumour of the testis and elevated plasma hCG concentration (> 10 IU $l^{-1}$) treated at the Department of Oncology, Aarhus University Hospital, and the Department of Oncology, Herlev Hospital, University of Copenhagen, were asked to enter the study. Entry criteria were the following: advanced non-seminomatous germ cell tumour of the testis classified as stage II or more or of extragonadal origin, age above 18 years, hCG > 10 IU $l^{-1}$ and signed informed consent.

The primary treatment comprised unilateral orchiectomy followed by chemotherapy with cisplatin 20 mg $m^{-2}$ days 1–5, etoposide 100 mg $m^{-2}$ days 1–5 and bleomycin 15 mg $m^{-2}$ on days 2, 9 and 16 (PEB) repeated every 3 weeks. Patients classified as having a poor prognosis received an intensified cisplatin dose of 40 mg $m^{-2}$. A standard course of treatment consisted of four treatment cycles. Observation and survival time were calculated from the start of treatment.

### Markers hCG, α-fetoprotein (AFP) and lactate dehydrogenase (LDH) were measured in all patients, but only hCG is considered in this report. The plasma disappearance of hCG was studied in two ways: (1) immunologically by measuring the plasma disappearance of total hCG and (2) by measuring the rate of plasma disappearance of $[^{125}I]$hCG.

Thyroid uptake of $^{125}I$ was prevented by giving 400 mg potassium iodide orally. $[^{125}I]$hCG (3 MBq) (Isopharma, Kjeller, Norway) was then given as a single intravenous bolus injection when a slow hCG decline was observed, usually between second and third treatment cycle. As the specific activity of the hCG was 50 MBq $mg^{-1}$ and the concentration was 3000 IU $mg^{-1}$, the quantity of additional hCG given was 180 IU. Following this single bolus injection of radiolabelled hCG, the $^{125}I$ concentration in plasma could be followed for up to 175 days. Total radiation dose following one injection was 0.03 mSv.

Radioactivity was measured in a well counter (COBRA II auto gamma, Packard, USA) set to detect within the energy range 15–75 keV. The background activity has been subtracted from all of the presented data. All plasma samples were counted at the same time to avoid calculated correction for the decay of $^{125}I$. The relative counting error was less than 5%. Results for $^{125}I$ concentration are presented as counts per minute for a 3-ml aliquot of plasma.

Total hCG was measured using a two-site immunometric assay specific for intact hCG using either time-resolved immunofluorometry with a detection limit of 5 pg $ml^{-1}$ (0.05 IU $l^{-1}$) (Madersbacher et al, 1993) or enzyme-linked immunosorbent assay (ELISA) with a detection limit of 2 IU $l^{-1}$ (215 pg $ml^{-1}$). The methods were calibrated using the Third International Standard (3 IS, 75/537; Kallner et al, 1993). Results for hCG concentrations are presented as IU per l (conversion ratio 1 IU = 108 ng). Plasma samples for $[^{125}I]$hCG and hCG measurements were obtained simultaneously two or three times weekly.

 *© 1999 Cancer Research Campaign*

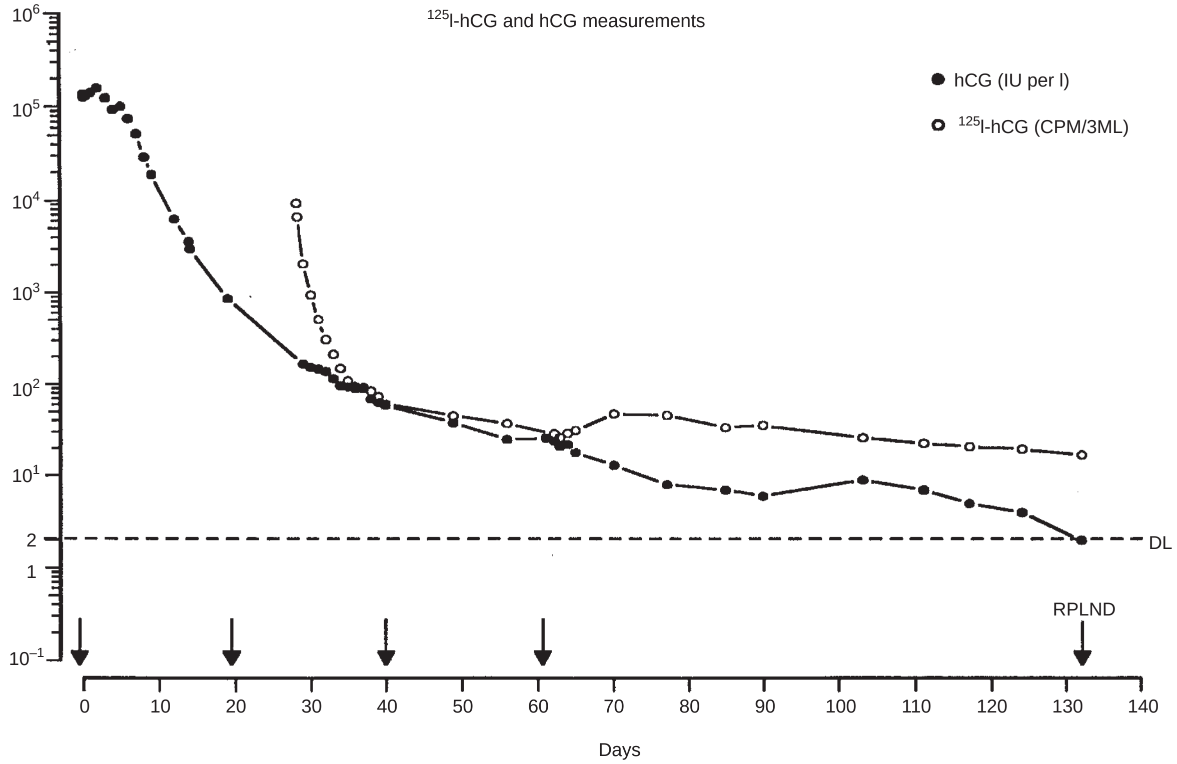

**Figure 2** Patient no. 2 with an initial hCG plasma concentration of 137 500 IU $l^{-1}$ receiving four cycles of high-dose PEB. Arrows indicate start of each cycle. [$^{125}$I]hCG was injected after completion of the second cycle of chemotherapy. The curves have a similar shape before start of the third cycle of chemotherapy. The patient only received four cycles of chemotherapy in spite of increased hCG plasma concentration and a prolonged half-life of hCG after the last cycle. At day 132, a residual retroperitoneal tumour was removed from retroperitoneum by retroperitoneal lymph node dissection (RPLND). Pathology showed necrosis without malignant cells. Thus, the patient achieved an SCR. The patient was alive with no evidence of disease 48+ months after initiation of chemotherapy

Initial and late hCG half-lives were calculated assuming a monoexponential disappearance using an iterative non-linear least square regression. Initial hCG half-life calculations were based on the median of five measurements (range 3–15), and only values on the declining part of the disappearance curve after the first cycle of chemotherapy were used, thus avoiding a potential marker surge. Late hCG half-life calculations were based on median of eight measurements (range 4–11) between the third and fourth cycles of chemotherapy.

All patients were given oral and written information about the study. The study was approved by the local ethics committees and the National Board of Health prior to inclusion of patients.

### RESULTS

A total of 27 patients entered the study, and the patients were observed until relapse and/or death or minimally 12 months after the start of chemotherapy.

Median age was 30 years (range 19–51 years). All patients had a non-seminomatous tumour, 26 of testicular origin and one of extragonadal origin. The median observation time from start of chemotherapy was 29 months (range 13–45 months) in the good-risk group and 29 months (range 2–48 months) in the poor-risk group. Twenty-two of the 27 patients were in continuous remission after a median time from start of treatment of 31 months (range 12–48 months). One patient (no. 27) with metastases to retroperitoneum, mediastinum, lungs and brain died during chemotherapy as a result of treatment-induced toxicity. Four patients had a relapse. Three of these have died as a result of progressive disease 9 months (no. 11), 13 months (no. 14) and 7 months (no. 22) after the start of chemotherapy. The remaining patient relapsed with increasing hCG concentrations 13 months after achieving a surgical complete remission (SCR). This patient had salvage chemotherapy and was alive with no evidence of active disease 44 months after start of primary chemotherapy.

### hCG

The median initial hCG for all patients was 1995 IU $l^{-1}$ (range 10–2 120 000 IU $l^{-1}$). In the 15 good-risk patients, the median initial hCG was 130 IU $l^{-1}$ (range 10–37 800 IU $l^{-1}$). In the 12 poor-risk patients, the initial median hCG was 108 757 IU $l^{-1}$ (range 64–2 120 000 IU $l^{-1}$). Eight had a hCG level above 40 000 IU $l^{-1}$ and six above 100 000 IU $l^{-1}$ before treatment.

A total of 1438 blood samples were taken. The plasma disappearance of hCG during chemotherapy showed a biphasic pattern, with an initial fast and a later slow component in all patients. In 18 patients with an initial hCG above 200 IU $l^{-1}$, hCG could be followed during the slow phase of plasma disappearance. Among these, 13 patients became marker negative and had no evidence of disease or were in continuous remission during follow-up. Figure 1 shows hCG measurements in these 13 patients. All had finished treatment on day 80. The points depict a biphasic pattern with an initial fast decline, corresponding to a half-life of 1.5–3.8 days to the level of 0.1% of the initial value. In the following prolonged hCG decline, each of these 13 patients had a half-life for hCG

© 1999 Cancer Research Campaign plasma disappearance longer than 3 days irrespective of the initial hCG concentration and risk status. Four of the five patients who did not achieve continuous remission had a similar hCG plasma disappearance pattern during chemotherapy, as described for patients who achieved a continuous remission. In the remaining patient (no. 22) who did not achieve continuous remission, hCG increased between the last two cycles of chemotherapy.

After injection of [$^{125}$I]hCG, the disappearance curve of [$^{125}$I]hCG showed a biphasic pattern with initial fast and a later slow component. The initial fast phase represents a situation of, simultaneously, distribution of [$^{125}$I]hCG into and elimination from the distribution volume. Thereafter, the plasma disappearance of [$^{125}$I]hCG mimics the final prolonged hCG decline. In the 13 patients from Figure 1 with an initial hCG > 200 IU $l^{-1}$, the final prolonged hCG decline followed the decline of [$^{125}$I]hCG for each individual patient, as exemplified in Figure 2. All of these 13 patients achieved continuous remission.

Four patients had a relapse. In two of these (nos. 11 and 14), the disappearance curves of hCG and [$^{125}$I]hCG showed similar paths until relapse was evidenced by a discrepancy between the curve of [$^{125}$I]hCG and the curve of immunologically measured hCG. In the third relapsing patient, the disappearance curves of hCG and [$^{125}$I]hCG also showed a similar path until hCG was below 2 IU $l^{-1}$. The patient relapsed with increasing hCG 13 months after start of chemotherapy. In the remaining patient (no. 22) who experienced relapse, the disappearance curves of hCG and [$^{125}$I]hCG did not have a similar shape.

By using the steep part of the hCG plasma disappearance curve, five good-risk patients (nos. 8, 10, 15, 18 and 19) and two poor-risk patients (nos. 4 and 9) had half-lives longer than 3 days, i.e. 3.2, 3.8, 4.7, 6.7, 3.6, 4.0 and 3.5 days respectively. All these patients achieved long-term remission after CR (five patients) or SCR (two patients). Three out of four patients, who had a relapse (nos. 6, 11 and 14) had half-lives shorter than 3 days, i.e. 2.8, 2.6 and 2.6 days. The remaining patient (no. 22) had a half-life of 3.7 days.

In 12 patients (nos. 2, 5, 7, 9, 11, 14, 17, 19, 20, 22, 26 and 27), hCG was elevated (> 2 IU $l^{-1}$) before the start of the fourth cycle of chemotherapy. The apparent half-lives of hCG between the third and fourth cycles of chemotherapy in these patients were between 7.4 and 17.0 days. Despite the prolonged half-life of hCG, 8 of these 12 patients were in continuous remission during follow-up. Three patients (nos. 11, 14 and 22) had a relapse and one patient (no. 27) died as a result of toxicity from treatment.

Four patients (nos. 2, 5, 7 and 26) had elevated hCG (> 2 IU per l) in plasma up to 150 days after the fourth cycle of chemotherapy. Three patients achieved an SCR (nos. 2, 7 and 26), and one continuous remission after a partial response (PR) (no. 5, see below). The disappearance of immunologically measured hCG and of [$^{125}$I]hCG in these four patients was similar, and the slow hCG decline was ascribed to a slow plasma disappearance and not to a continuous formation of hCG from vital tumour cells. All four patients became hCG negative (< 2 IU $l^{-1}$) without additional chemotherapy and were in continuous remission for 48+, 44+, 39+ and 12+ months, respectively, after initiation of chemotherapy.

When the present study was initiated, the treatment strategy in Denmark was to give one or two extra cycles of chemotherapy if hCG was not normalized and decreased with a half-life longer than 3 days before the fourth treatment cycle. Using this recommended treatment strategy, eight poor-risk patients (nos. 2, 5, 7, 9, 11, 14, 22 and 26) and one good-risk patients (no. 17) were considered for additional/intensified chemotherapy. These patients all had an elevated plasma concentration and a prolonged half-life of hCG when starting the fourth cycle of chemotherapy as well as after completing the four treatment cycles. In patient no. 22, who progressed during chemotherapy, the disappearance curves for hCG and [$^{125}$I]hCG deviated. This patient refused further chemotherapy and died 7 months after initiation of chemotherapy. In the other eight patients, the disappearance curves for hCG and [$^{125}$I]hCG had similar paths before and after the four cycles of chemotherapy. Hence, no further chemotherapy was given. The good-risk patient (no. 17) achieved an SCR and was alive with no evidence of disease 22+ months after initiation of chemotherapy. Similarly, four out of the seven poor-risk patients (nos. 2, 7, 9 and 26) achieved an SCR. They were all alive with no evidence of disease 48+, 39+, 36+ and 12+ months, respectively, after initiation of chemotherapy. One of the poor-risk patients (no. 5) had metastases to retroperitoneal lymph nodes, lungs, liver and spleen. He achieved a partial response. Residual tumours in lungs, liver and spleen were inaccessible for radical surgery. However, multiple biopsies revealed only necrosis and fibrosis. This patient was alive with further and continuous remission 44+ months after initiation of chemotherapy.

The remaining two poor-risk patients (nos. 11 and 14) considered for additional chemotherapy had a relapse with increasing hCG concentrations shortly after completion of the fourth cycle of chemotherapy. This was evidenced by a discrepancy between the disappearance of [$^{125}$I]hCG and total hCG. One of the patients refused further chemotherapy and died after 9 months. The other patient had supralethal chemotherapy followed by autologous bone marrow transplantation. He relapsed again, with increasing hCG plasma concentration, 30 days after treatment and died 13 months after initiation of the primary chemotherapy.

### DISCUSSION

A half-life of hCG longer than 3 days during chemotherapy for testicular cancer has been used to identify patients with a high risk of relapse and who are thus candidates for intensified therapy (Toner et al, 1990; Motzer et al, 1992; Bosl, 1993; Motzer et al, 1993; Bosl and Chaganti, 1994; Murphy et al, 1994; Gerl et al, 1996; Bosl and Motzer, 1997). On the other hand, Horwich & Peckham (1984), Stevens et al (1995), de Wit et al (1997) and Zon et al (1998) did not find any association between response to chemotherapy and a prolonged half-life of hCG longer than 3 days.

The plasma disappearance of hCG may depend on treatment-induced toxicity on elimination organs, changes in volume of distribution of hCG caused by loss of weight, disease-induced changes in elimination, receptor or unspecific binding of hCG in different tissues or varying production or release of hCG from tumour cells during therapy. Because of these factors, we considered the commonly used monoexponential model for the disappearance of hCG with an acceptable half-life of less than 3 days to be too simplistic.

The present study indicates that the plasma disappearance of hCG during chemotherapy can be quite different from the commonly used monoexponential model. In all patients, a biphasic plasma disappearance with an initial fast and a late slow phase was observed. Serial measurements of hCG from 13 patients receiving chemotherapy showed a half-life of hCG between the third and fourth treatment cycle longer than 3 days. Despite this ‘prolonged’ half-life, all 13 patients were in continuous remission ranging from 12+ to 48+ months after completion of chemotherapy. This is in

 © 1999 Cancer Research Campaign accordance with data reported by Andreyev et al (1993) and Zon et al (1998) and substantiates our observation that a slow half-life of hCG late during or after treatment is not necessarily associated with vital hCG-producing tumour cells. Others have used the initial half-life to select patients for intensified chemotherapy (Toner et al, 1990; Bosl, 1993; Motzer et al, 1993). In our study, an initial half-life longer than 3 days did not predict the outcome of chemotherapy. Thus, seven patients who achieved long-term remission had initial half-lives longer than 3 days. In contrast, we found an initial half-life shorter than 3 days in three out of four patients who had a relapse.

To address the question of whether a slow decline of hCG during chemotherapy is caused by continuous production of hCG from vital tumour cells or a slow plasma disappearance, we used [$^{125}$I]hCG as an elimination marker. Monitoring [$^{125}$I]hCG gave us a model for the disappearance of hCG under the assumption of no production of hCG. This allowed us to consider the plasma disappearance of hCG regardless of treatment or other interfering factors. If the curve of immunologically measured hCG showed a slow disappearance but was similar in shape to the disappearance of [$^{125}$I]hCG, we interpreted the prolonged half-life as caused by a slow plasma disappearance rather than a production of hCG from vital tumour cells. This seems to be in accordance with the subsequent outcome for the majority of patients, although it should be emphasized that this pattern does not ensure long-term survival.

The study suggests that the concept of a fixed half-life or apparent half-life of hCG based on immunological hCG measurements during chemotherapy may be misleading. Thus, a threshold value of a hCG half-life of 3 days may lead to serious mistakes when deciding further treatment. We recommend that decision on further treatment should be based on inspection of a curve of frequently measured plasma hCG concentrations rather than on calculation of hCG half-lives. The use of [$^{125}$I]hCG plasma disappearance curves may be helpful in the interpretation of the results. If the disappearance curves correspond, a so-called prolonged half-life of hCG and persistence of hCG in plasma does not by itself justify a change of the treatment strategy.

### ABBREVIATIONS

hCG, gonadotropin chorionic (human); NED, no evidence of disease; CR, complete remission; SCR, surgical complete remission (defined as: residual tumour removed by surgery and histopathological examination including microscopy showing only necrosis or fibrosis or mature teratoma. If mature teratoma is found in the residual tumour, the operation should be radical for fulfilling the criteria of an SCR); PR, partial remission; PD, progressive disease; RPLND, retroperitoneal lymph node dissection; CD, conventional dose chemotherapy; HD, high-dose chemotherapy; DL, detection limit.

### ACKNOWLEDGEMENTS

We thank Professor William E Watson, Department of Physiology, University of Edinburgh, for careful reviewing and criticism of the manuscript. This study has been supported by grants from: Danish Cancer Society, Grosserer Valdemar Foersom og hustru Thyra Foersom, født Otto's fond, Clinical Research Unit Aarhus University Hospital, Clinical Research Unit Herlev Hospital, University of Copenhagen, Ingeniør August Frederik Wedell Erichens Legat and Direktør Jacob Madsens og Hustru Olga Madsens Fond.

### REFERENCES

Andreyv HJN, Dearnaley DP and Horwich A (1993) Testicular non-seminoma with high serum human chorionic gonadotrophin: the trophoblastic teratoma syndrome. *Diagnos Oncol* 67–71

Bosl GJ (1993) Prognostic factors for metastatic testicular germ cell tumours: the Memorial Sloan–Kettering cancer model. *Eur Urol* **23**: 182–187

Bosl GJ and Chaganti RSK (1994) The use of tumor markers in germ cell malignancies. *Hematol Oncol Clin N Am* **8**: 573–587

Bosl GJ and Motzer RJ (1997) Testicular germ-cell cancer. *N Engl J Med* **337**: 242–253

De Wit R, Sylvester R, Tsitsa C, De Mulder PH, Sleyfer DT, Ten Bokkel Huinink WW, Kaye SB, van Oosterom AT, Boven E, Vermeylen K and Stoter G (1997) Tumour marker concentration at the start of chemotherapy is a stronger predictor of treatment failure than marker half-life: a study in patients with disseminated non-seminomatous testicular cancer. *Br J Cancer* **75**: 432–435

Gerl A, Lamerz R, Clemm C, Mann K, Hartenstein R and Wilmanns W (1996) Does serum tumor marker half-life complement pretreatment risk stratification in metastatic nonseminomatous germ cell tumors? *Clin Cancer Res* **2**: 1565–1570

Horwich A and Peckham MJ (1984) Serum tumour marker regression rate following chemotherapy for malignant teratoma. *Eur J Cancer Clin Oncol* **20**: 1463–1470

Kallner A, Magid E and Ritchie R (1993) Improvement of comparability and compatibility of laboratory assay results in life sciences. *Scand J Clin Lab Invest* (suppl.) **53**: 42–139

Klein EA (1993) Tumor markers in testis cancer. *Urol Clin N Am* **20**: 67–73

Madersbacher S, Stulnig T, Huber LA, Schonitzer D, Dirnhofer S, Wick G and Berger P (1993) Serum glycoprotein hormones and their free alpha-subunit in a healthy elderly population selected according to the SENIEUR protocol. Analyses with ultrasensitive time resolved fluoroimmunoassays. *Mech Age Dev* **71**: 223–233

Motzer RJ, Gulati SC, Crown JP, Weisen S, Doherty M, Herr H, Fair W, Sheinfeld J, Sogani P, Russo P and et al (1992) High-dose chemotherapy and autologous bone marrow rescue for patients with refractory germ cell tumors. Early intervention is better tolerated. *Cancer* **69**: 550–556

Motzer RJ, Mazumdar M, Gulati SC, Bajorin DF, Lyn P, Vlamis V and Bosl GJ (1993) Phase II trial of high-dose carboplatin and etoposide with autologous bone marrow transplantation in first-line therapy for patients with poor-risk germ cell tumors. *J Natl Cancer Inst* **85**: 1828–1835

Murphy BA, Motzer RJ, Mazumdar M, Vlamis V, Nisselbaum J, Bajorin D and Bosl GJ (1994) Serum tumor marker decline is an early predictor of treatment outcome in germ cell tumor patients treated with cisplatin and ifosfamide salvage chemotherapy. *Cancer* **73**: 2520–2526

Stevens MJ, Norman AR, Dearnaley DP and Horwich A (1995) Prognostic significance of early serum tumor marker half-life in metastatic testicular teratoma. *J Clin Oncol* **13**: 87–92

Toner GC, Geller NL, Tan C, Nisselbaum J and Bosl GJ (1990) Serum tumor marker half-life during chemotherapy allows early prediction of complete response and survival in nonseminomatous germ cell tumors. *Cancer Res* **50**: 5904–5910

Zon RT, Nichols C and Einhorn LH (1998) Management strategies and outcomes of germ cell tumor patients with very high human chorionic gonadotropin levels. *J Clin Oncol* **16**: 1294–1297

© 1999 Cancer Research Campaign 
